# Loss of *TP53* cooperates with c-MET overexpression to drive hepatocarcinogenesis

**DOI:** 10.1038/s41419-023-05958-y

**Published:** 2023-07-27

**Authors:** Yi Zhou, Guofei Cui, Hongwei Xu, Joanne Chun, Doris Yang, Zheng Zhang, Lihui Yang, Jingxiao Wang, Meijuan Wan, Diego F. Calvisi, Shumei Lin, Xin Chen, Haichuan Wang

**Affiliations:** 1 Department of Infectious Diseases, The First Affiliated Hospital of Xi’an Jiaotong University, Xi’an, China; 2 Department of Bioengineering and Therapeutic Sciences and Liver Center, University of California, San Francisco, San Francisco, CA USA; 3 Liver Cancer Laboratory, University of Hawaii Cancer Center, Honolulu, HI USA; 4 Division of Liver Surgery, Department of General Surgery, West China Hospital, Sichuan University, Chengdu, China; 5 Laboratory of Liver Surgery, West China Hospital, Sichuan University, Chengdu, China; 6 Beijing University of Chinese Medicine, Beijing, China; 7 School of Life Sciences, Beijing, University of Chinese Medicine, Beijing, China; 8 Institute of Pathology, University of Regensburg, Regensburg, 93053 Germany

**Keywords:** Liver cancer, Experimental models of disease

## Abstract

Hepatocellular carcinoma (HCC) is a deadly malignancy with high genetic heterogeneity. *TP53* mutation and c-MET activation are frequent events in human HCCs. Here, we discovered that the simultaneous mutations in *TP53* and activation of c-MET occur in ~20% of human HCCs, and these patients show a poor prognosis. Importantly, we found that concomitant deletion of *Trp53* and overexpression of c-MET (c-MET/sgp53) in the mouse liver led to HCC formation in vivo. Consistent with human HCCs, RNAseq showed that c-MET/sgp53 mouse HCCs were characterized by activated c-MET and Ras/MAPK cascades and increased tumor cell proliferation. Subsequently, a stably passaged cell line derived from a c-MET/sgp53 HCC and corresponding subcutaneous xenografts were generated. Also, in silico analysis suggested that the MEK inhibitor trametinib has a higher inhibition score in *TP53* null human HCC cell lines, which was validated experimentally. We consistently found that trametinib effectively inhibited the growth of c-MET/sgp53 HCC cells and xenografts, supporting the possible usefulness of this drug for treating human HCCs with *TP53*-null mutations. Altogether, our study demonstrates that loss of *TP53* cooperates with c-MET to drive hepatocarcinogenesis in vivo. The c-MET/sgp53 mouse model and derived HCC cell lines represent novel and useful preclinical tools to study hepatocarcinogenesis in the *TP53 null* background.

## Introduction

Primary liver cancer is one of the most common malignant tumors worldwide. Hepatocellular carcinoma (HCC) is the predominant tumor entity, accounting for 75–85% of the cases [1]. HCC is often associated with chronic liver disease, which causes liver cell death, inflammation, oxidative stress, and fibrosis. At the molecular level, HCC is driven by heterogeneous signaling pathways that produce DNA damage to liver cells, resulting in resistance to cell death, proliferation, and, eventually, tumorigenesis [2]. Though targeted therapies and immunization treatments for HCC have been extensively investigated, only a few multi-kinase and immune checkpoint inhibitors have been approved for advanced HCCs [3]. Therefore, identifying the specific aberrant molecular pathways that drive hepatocarcinogenesis and generating preclinical models to explore targeted treatments are essential for HCC patients.

The *TP53* (p53) gene is one of the most critical tumor suppressors. The p53 protein encoded by *TP53* is involved in various pathways to regulate multiple processes, such as metabolism, DNA damage repair, cell cycle arrest, and apoptosis. As in other cancer types, *TP53* mutation is also one of the main genetic variations in HCC. Accounting for about 30% of HCC cases, *TP53* inactivating mutations contribute to HCC initiation and progression [4–6]. In addition, reactivating p53 in tumors with p53 inactivation can induce tumor stabilization or regression [7, 8]. Therefore, drugs targeting p53 loss of function could represent a promising approach for HCC treatment.

*MET* (c-MET) is a protooncogene that encodes the hepatocyte growth factor (HGF) receptor. Binding to HGF, c-MET activates multiple downstream targets, such as the RAS/MAPK and phosphoinositide 3-kinase (PI3K)/AKT pathways, to drive tumor invasion and metastasis [9]. Previous studies have shown that c-MET activation occurs in 20–44% of HCC patients, and increased expression of c-MET is associated with poor tumor differentiation, augmented intrahepatic metastases, and poor prognosis [10, 11]. However, c-MET alone cannot induce HCC in mice but requires the presence of additional molecular events, such as AKT overexpression, β-Catenin activation, or loss of the PTEN oncosuppressor to drive liver malignant transformation [12–14].

Gene expression analysis of HCC specimens from The Cancer Genome Atlas-Liver Hepatocellular Carcinoma (TCGA) database suggests that samples with *TP53* mutations are more likely to show upregulation of the c-MET signature. Specifically, about 22% of the HCC patients have both c-MET activation features and *TP53* mutations. Clinically, these HCCs are associated with poor patients’ prognoses. Therefore, we speculate that *TP53* mutations can synergize with c-MET activation to induce hepatocyte malignant transformation. In this study, we established and characterized a novel murine HCC model induced by overexpression of c-MET and CRISPR-Cas9 mediated knockout of *Trp53* (encoding mouse p53). In addition, we applied this model for therapeutic testing.

## Results

### c-MET activation and *TP53* mutations occur concomitantly in a subset of human HCCs

**Fig. 1:**
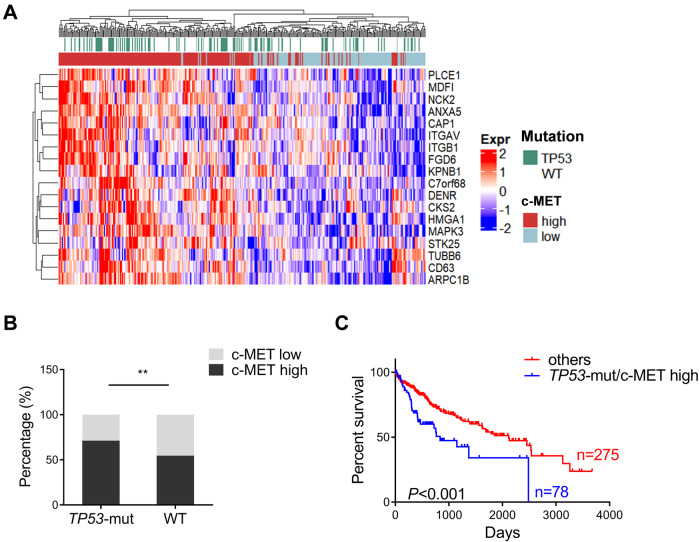
A subset of human HCC samples with c-MET activation and *TP53* mutations based on the TCGA dataset. **A** Heatmap of the human HCC samples depicting *TP53* mutations and c-MET activation. **B** c-MET signature is enriched in *TP53* mutant human HCC samples. **C** Survival curves of patients harboring concomitant *TP53* mutation and c-MET activation, indicating a poor prognosis compared to other patients (includes *TP53* mutant/c-MET low, *TP53* WT/c-MET low, and *TP53* WT/c-MET high HCCs). Data are shown as mean ± SD. **B** Student’s *t*-test: **P < 0.01; **C** Log-rank (Mantel–Cox) test. WT wild-type.

c-MET overexpression and *TP53* mutation are two frequent alterations reported in human HCC. To investigate the profile of these genetic aberrations, we analyzed the expression level of c-MET and *TP53* mutation status based on The Cancer Genome Atlas-Liver Hepatocellular Carcinoma (TCGA LIHC) human HCC dataset [15]. Based on the sequencing data from TCGA, the types of *TP53* mutation include truncating mutations, splice mutations, inframe mutations, and missense mutations. The details about *TP53* mutations are shown in Fig. S1. As the levels of c-MET protein expression are not correlated with c-MET activation status, the c-MET signature score was applied, which was identified using the KAPOSI_LIVER_CANCER_MET_UP gene set [16], described previously [17]. First, we determined the c-MET activation signature in *TP53* mutant and *TP53* wild-type samples. The results showed that the c-MET signature was enriched in *TP53* mutant human HCC samples (Fig. 1A). Indeed, ~71% of *TP53* mutant HCCs (79/111) in the TCGA dataset displayed the activated c-MET signature. In comparison, only 55% of *TP53* wild-type HCCs (136/249) exhibited high c-MET expression (Fig. 1B). Further analysis showed that in human HCC, ~22% of specimens harbor concomitant *TP53* mutation and c-MET activation. Importantly, these HCC patients exhibited the shortest overall survival (Fig. 1C). The overall survival from *TP53* mutant c-MET high or low HCC patients was compared with the *TP53* wild-type group, and no significant differences were observed (Fig. S2).

In summary, *TP53* mutation and c-MET activation co-occur in a subset of human HCC patients with poor prognoses.

### Deletion of *Trp53* synergizes with activated c-MET to promote HCC formation in mice

**Fig. 2:**
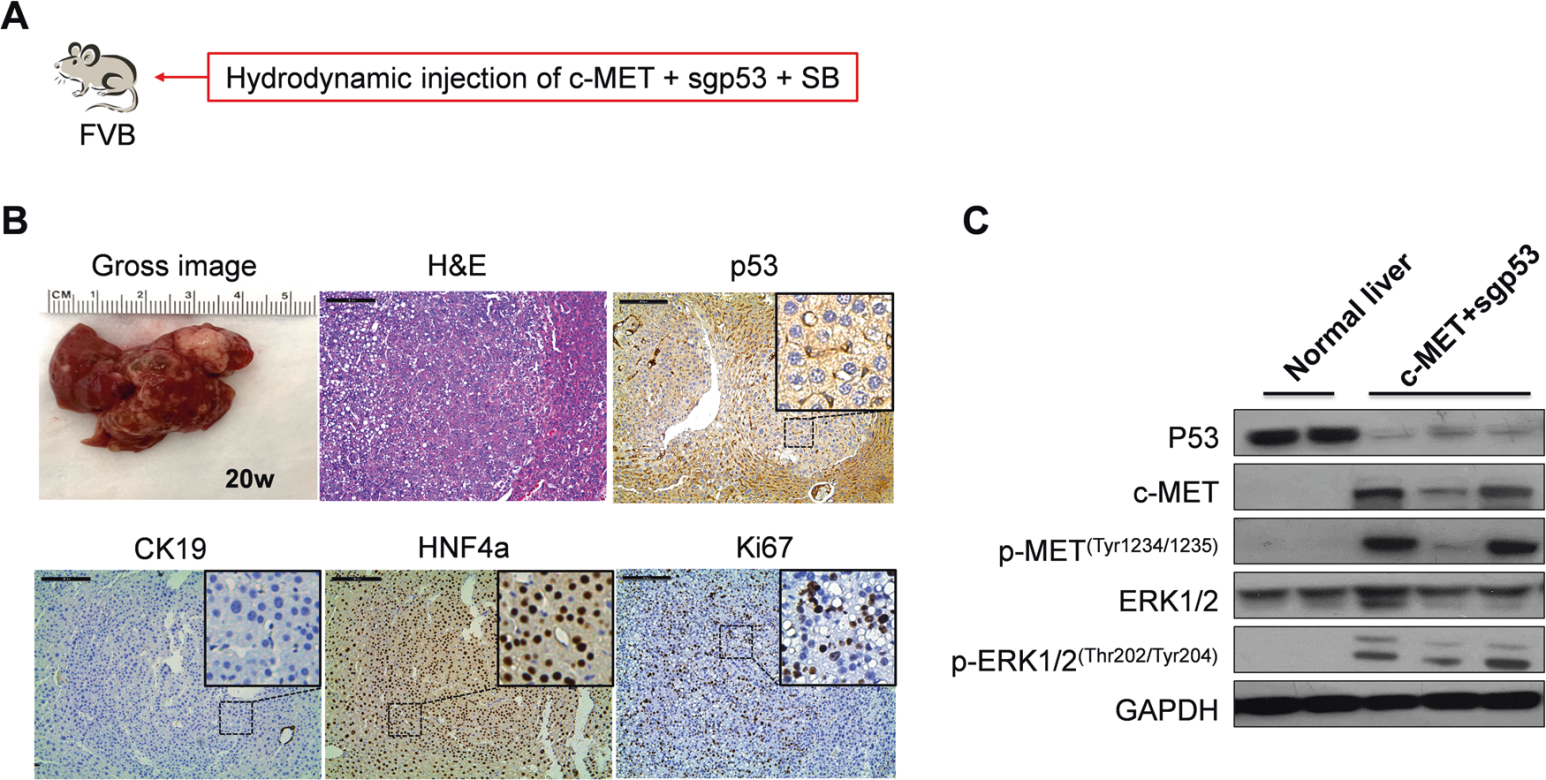
Loss of *TP53* synergizes with c-MET to promote hepatocarcinogenesis in mice. **A** Study design. **B** Gross images, H&E staining, and immunohistochemical staining of p53, CK19, HNF4a, and Ki67 in tumor lesions from c-MET/sgp53 mouse liver. **C** Western blotting analysis of lysates from normal liver and c-MET/sgp53 mouse HCCs. Scale bar: 200 μm (100×). H&E hematoxylin and eosin staining, CK19 cytokeratin 19, HNF4a hepatocyte nuclear factor 4α.

Our previous work suggested that long-term overexpression of c-MET alone by hydrodynamic injection in the mouse liver does not trigger liver tumor development while giving rise to dysplastic foci [17, 18]. Similarly, sporadic loss of *TP53* in the mouse liver does not induce liver tumor formation [19]. Therefore, we hypothesized that the simultaneous deletion of *TP53* and expression of c-MET might lead to hepatocarcinogenesis in mice. Using the CRISPR–Cas9-mediated gene editing method, we stably deleted *Trp53* in mouse hepatocytes using the pX330-sgP53 plasmid [19]. The pX330-sgp53 construct was co-expressed with the pT3-EF1α-c-MET and pCMV-SB constructs via hydrodynamic tail-vein injection (c-MET/sgp53) (Fig. 2A). Consistent with our hypothesis, c-MET/sgp53 combination induced liver tumor formation in vivo (Table S2). Gross tumor nodules were observed in the mouse liver between 17 and 30 weeks post-injection. Tumors varied in size, and histological evaluation revealed that the tumor lesions were consistent with well-differentiated HCC. No extrahepatic metastases developed in these mice. In addition, tumors were characterized by positive HNF-4a immunoreactivity, negative CK19 staining, and high Ki67 immunolabeling, indicating their hepatocellular nature and robust proliferative activity (Fig. 2B). Gene expression analysis demonstrated the upregulation of HCC-related genes, including *Afp*, *Gpc3*, and *Prom1*, as well as genes associated with cell proliferation, such as *Ccnb1*, *Ccne1*, *Cdk6, Bub1*, and *Mki67* in c-MET/sgp53 tumors (Fig. S3). Furthermore, immunohistochemical (IHC) staining and Western blotting verified the efficient deletion of p53 in the HCC lesions. Western blot analysis also revealed the overexpression of c-MET and the corresponding phosphorylation/activation of c-MET (p-MET). Moreover, c-MET/sgp53 tumor lesions exhibited phosphorylation/activation of the ERK signaling (p-ERK) (Fig. 2C, Fig. S4, and Fig. S14). To further validate the effective deletion of the *Trp53* gene in c-MET/sgp53 HCCs, we performed genome sequencing on mouse *Trp53* alleles in tumor nodules. The Sanger sequencing confirmed the nucleotide deletions of *Trp53* on its genomic locus (Fig. S5).

### Activation of the RAS/MAPK cascade in c-MET/sgp53 lesions

**Fig. 3:**
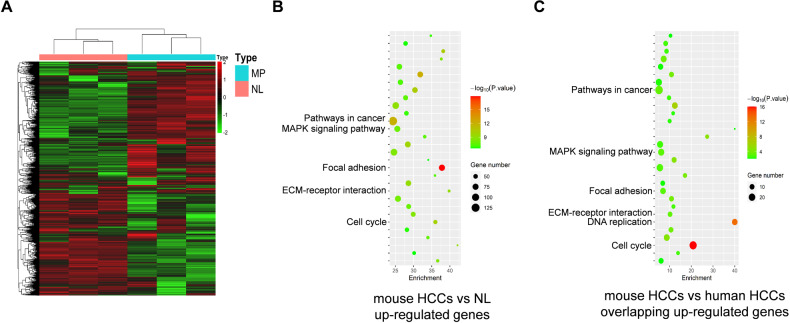
RNA sequencing data show the activation of the MAPK signaling pathway in c-MET/sgp53 HCCs. **A** Genetic dissimilarity among the samples in c-MET/sgp53 HCCs and normal liver as demonstrated by the heatmap. **B** KEGG analysis of upregulated DEGs in the c-MET/sgp53 HCCs as compared to normal livers. **C** KEGG analysis of overlapping up-regulated DEGs in c-MET/sgp53 HCCs and c-MET-high/*TP53*-null human HCCs. NL normal livers, ST surrounding tissues, DEG differentially expressed genes.

To fully understand the molecular effectors of c-MET/sgp53 HCCs, we performed RNA sequencing of tissues from normal mouse livers (*n* = 3) and c-MET/sgp53 liver tumors (*n* = 3). The heatmap of the gene list showed genetic dissimilarities between the two groups (Fig. 3A). The differentially expressed genes (DEG) analysis identified 2651 genes that were significantly upregulated in c-MET/sgp53 HCC samples (fold change, >1.5; *P* adj < 0.05) (Fig. S6A and Table S1). The Kyoto Encyclopedia of Genes and Genomes (KEGG) analysis revealed that the upregulated genes were enriched in tumor-associated pathways, including pathways in cancer, focal adhesion, ECM–receptor interaction, and cell cycle, in c-MET/sgp53 HCCs versus normal livers (Fig. 3B and Fig. S6B). Interestingly, KEGG analysis showed DEGs significantly enriched in the MAPK signaling pathway (Fig. 3B and Fig. S6B). The corresponding heatmap showed that the differential expression of the MAPK cascade correlated genes in c-MET/sgp53 mouse HCCs compared to normal livers (Fig. S7 and Table S3).

To further model c-MET/sgp53 mouse liver tumors to human HCCs, we analyzed a subset of human HCC samples harboring concomitant c-MET activation and *TP53* deletion using the TCGA-LIHC dataset, which occurred in 23/360 HCC specimens (Fig. S8A). Multidimensional scaling (MDS) analysis showed genetic dissimilarity among the samples in c-MET-high/*TP53*-null human HCCS and surrounding tissues (Fig. S8B). Compared to surrounding liver tissues, 2817 genes were upregulated (fold change, >1.5; *P* adj < 0.05) in c-MET-high/*TP53*-null HCC samples, among which 521 genes were also upregulated in c-MET/sgp53 mouse HCCs (Fig. S6A). In particular, the upregulated genes were enriched in cancer-related pathways (Fig. S8C). In addition, the 521 overlapping upregulated genes were mainly enriched in DNA replication, cell cycle, pathways in cancer, focal adhesion, and ECM–receptor interaction. Notably, the overlapping DGEs were also significantly enriched in the MAPK pathway (Fig. 3C and Fig. S6C).

In summary, c-MET/sgp53 mouse HCC tissues exhibit distinct gene expression profiles that partially overlap with human HCCs harboring concomitant c-MET activation and *TP53* deletion. Furthermore, both mouse and human HCCs with c-MET activation and *TP53* loss show the activation of the MAPK cascade.

### Establishment and application of the murine HCC cell line derived from c-MET/sgp53 HCC

**Fig. 4:**
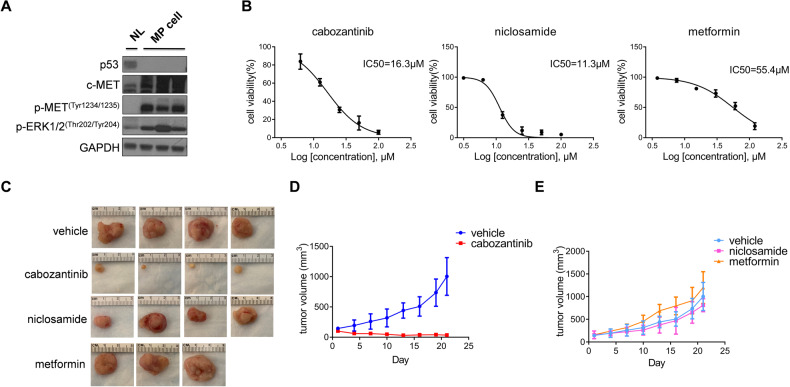
Establishment of a stably passaged cell line (MP) derived from c-MET/sgp53 HCC. **A** Western blotting analysis of lysates from normal liver and MP cells. **B** Cell viability analysis for MP cells treated with cabozantinib, niclosamide, and metformin. **C** Gross images of MP-derived xenografts from mice treated with cabozantinib, niclosamide, metformin, and vehicles. **D** Tumor volume of mice treated with cabozantinib and vehicles. **D** Tumor volume of mice treated with niclosamide, metformin, and vehicles. Data are shown as mean ± SD.

To investigate the potential therapeutic strategies for c-MET/sgp53 tumors, we developed a stably passaged cell line (MP cells) derived from a c-MET/sgp53 HCC by serial passaging of tumor cells from mouse to mouse (Figs. S9 and S10). Western blot analysis confirmed the deletion of p53 and the upregulation of p-MET and p-ERK protein in MP cells (Fig. 4A). To verify the usefulness of this tumor cell line, FVB/N mice were injected in the flank with the MP cells. Two weeks after injection, the subcutaneous tumor model was successfully established, supporting the oncogenic potential of this cell line.

Next, to illustrate the gene expression patterns of stably passaged c-MET/sgp53 tumor cells (MP cells), we also performed RNA-Seq experiments on MP cells. Normal liver tissues from FVB/N mice were used as the control. The DEGs analysis identified 4697 significantly upregulated genes (fold change, >1.5; *P* adj < 0.05) in MP cells (1578 genes overlapped with the MP tumors) (Fig. S11A). KEGG analysis revealed that pathways enriched in the initial tumors, including pathways in cancer, focal adhesion, ECM–receptor interaction, and cell cycle, were also enriched in MP cells. The MAPK pathway was consistently upregulated in MP cells (Fig. S11B, C). Notably, the DEGs upregulated in MP tumors but not in MP cells were enriched in immune-related pathways, such as antigen processing and presentation, cell adhesion molecules, and cytokine receptor interaction, suggesting that MP cell lines are less suitable for tumor immune-related studies (Fig. S11D).

Cabozantinib is an FDA-approved drug for HCC treatment, and it inhibits HCC growth by targeting the activated c-MET pathway [20, 21]. Therefore, we hypothesized that MP cells would be sensitive to cabozantinib treatment. To test this hypothesis, we treated MP cells with cabozantinib in culture and found an IC50 value around 16 μM (Fig. 4B), consistent with human HCC cell lines sensitive to cabozantinib [20]. Next, we established a xenograft model to investigate MP cell sensitivities in vivo further. Tumor-bearing mice were treated with a daily dose of cabozantinib (60 mg/kg/day) or vehicle control. After 3 weeks of treatment, cabozantinib-treated mice showed a robust reduction in tumor growth compared to vehicle-treated mice (Fig. 4C, D) (Table S4). Altogether, these data support the usefulness of the MP cell line and corresponding subcutaneous xenografts for investigating therapeutic strategies targeting p53-defective HCC and/or c-MET-activated HCC.

As the loss of *TP53* is one of the most frequent genetic events in human HCCs, we applied this unique murine HCC cell line to study drugs that have shown effectiveness against p53-defective tumors. Niclosamide and metformin have been reported to inhibit the growth of xenografts from p53-defective human cancer cells [22, 23]. Nevertheless, clinical evidence supporting the effectiveness of these drugs for *TP53* null human cancers is lacking. We found that both drugs could inhibit MP cell growth in vitro (Fig. 4B). However, in MP xenograft models, neither niclosamide nor metformin showed any efficacy against MP cells (Fig. 4C, E) (Table S4). The results imply that these drugs are unlikely to be useful against human HCCs with *TP53* mutations.

### Trametinib is effective against the *TP53*-null Hep3B human HCC cell line

**Fig. 5:**
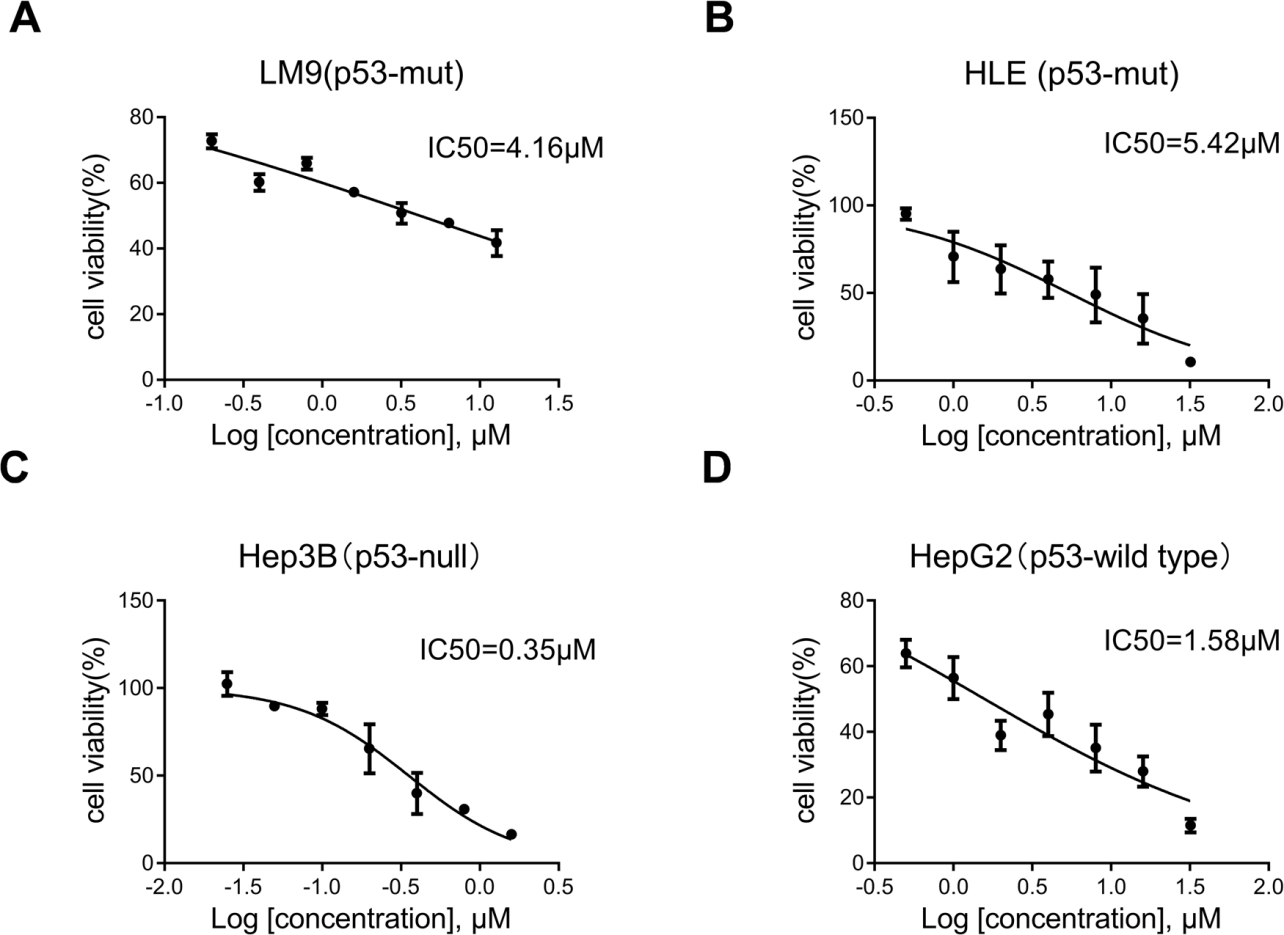
Treatment with trametinib has a lower IC50 in the *TP53*-null human HCC cells in vitro. Three human HCC cell lines **A** LM9 (p53-mutant, c-MET activated), **B** HLE (p53-mutant, c-MET activated), **C** Hep3B (p53-null, c-MET inactivated), and one hepatoblastoma cell line **D** HepG2 (p53-wild type, c-MET inactivated) were treated with escalating concentrations of trametinib for 48 h, and IC50 values were calculated.

As the bioinformatics analysis indicated (Fig. 3B, C), the MAPK signaling pathway was upregulated in c-MET/sgp53 mouse HCCs and c-MET-high/*TP53*-null human HCCs. Consistently, we searched drug-response information of the human HCC cell lines in the Genomics of Drug Sensitivity in Cancer database (www.cancerRxgene.org). The results suggested that the *TP53*-null HCC cell line (Hep3B) has higher sensitivity to multiple MEK inhibitors treatment than p53-mutant and p53-wild-type human HCC cell lines (Fig. S12). Accordingly, we validated the in vitro efficacy of the MEK inhibitor trametinib in the LM9 (p53-mutant), HLE (p53-mutant), and Hep3B (p53-null) human HCC cell lines and the HepG2 (p53-wild type) hepatoblastoma cell line. As expected, trametinib treatment had a lower IC50 value in Hep3B than the other HCC and hepatoblastoma cell lines (Fig. 5), suggesting that inhibition of the MAPK pathway might be a potential therapeutic strategy against *TP53*-null HCCs.

### Trametinib inhibits the growth of c-MET/sgp53 HCC cells and xenografts

**Fig. 6:**
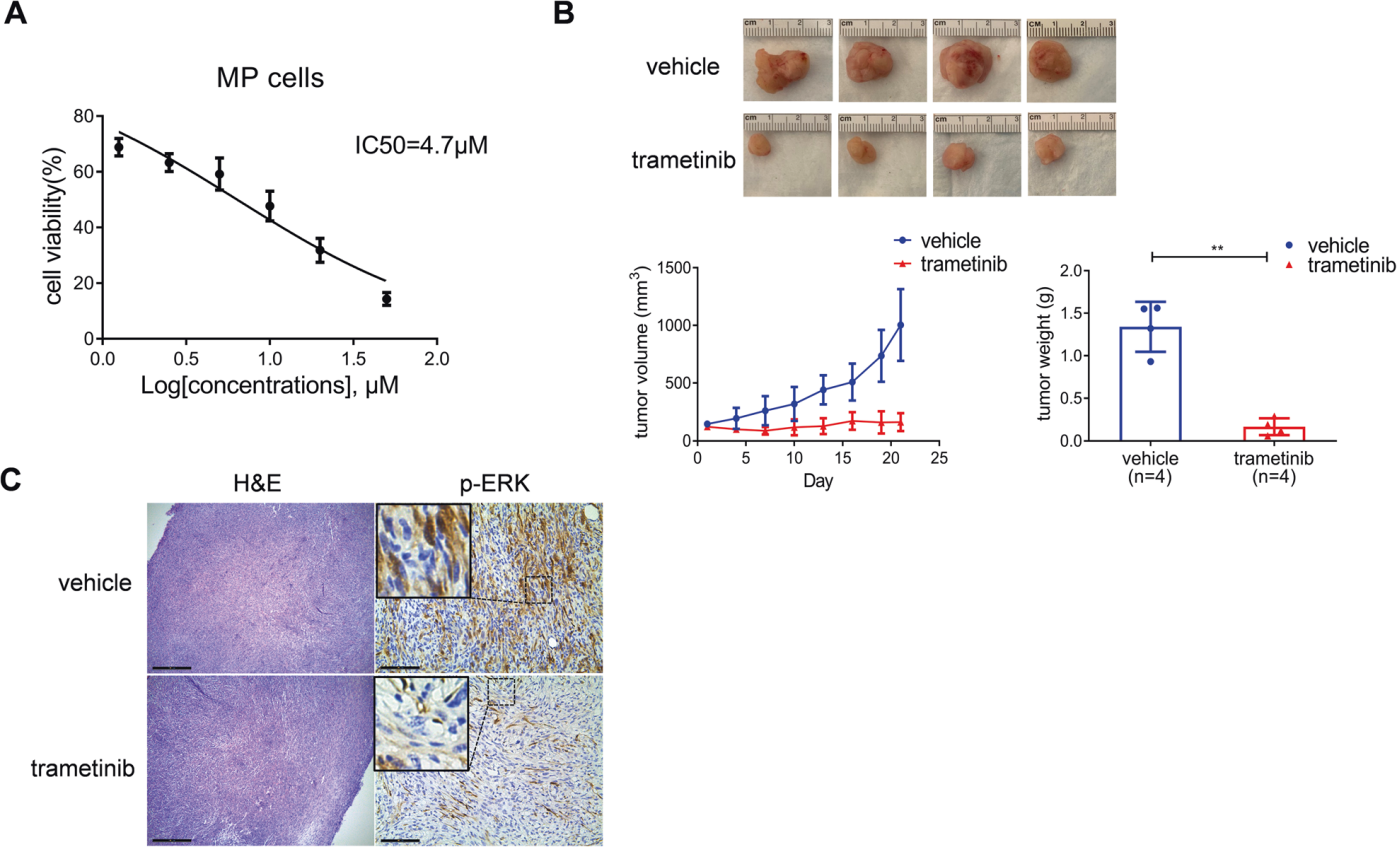
Trametinib inhibits the growth of c-MET/sgp53 HCC cells and their xenografts. **A** Cell viability analysis of MP cells treated with trametinib. **B** Gross images, tumor volume, and tumor weight of MP-derived xenografts from mice treated with trametinib and vehicles. **C** H&E staining and immunohistochemical staining of p-ERK in xenografts from mice treated with trametinib and vehicles. Scale bar: H&E, 500 μm (40×); p-ERK, 200 μm (100×). Student’s *t*-test: ***P < 0.001. H&E hematoxylin and eosin staining.

**Fig. 7:**
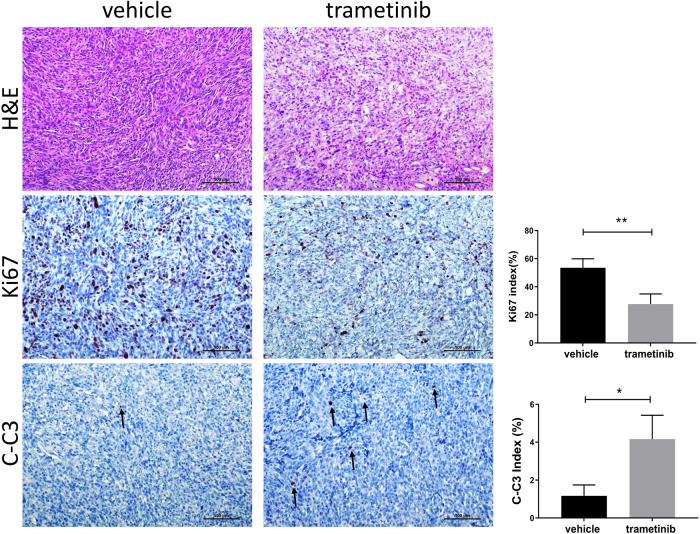
Trametinib treatment decreases proliferation and induces apoptosis in c-MET/sgp53 HCC cell xenografts. Ki67 and cleaved-caspase 3 (C-C3) indices, markers of proliferation and apoptosis, respectively, were assessed by evaluating the number of Ki-67 and C-C3 positive cells in at least 3000 tumor cells per sample. Three mice per group were analyzed. H&E hematoxylin and eosin staining. Scale bar: 100 µm (200×). Student’s *t*-test: **P < 0.001; *P < 0.05.

Based on the in vitro studies, we investigated whether the MEK inhibitor effectively inhibits *TP53*-defective tumor growth. We selected trametinib, an FDA-approved MEK inhibitor used to treat BRAF (V600E) mutant metastatic melanoma [24]. First, we administered trametinib to MP cells in culture. We found that trametinib effectively inhibits MP cell growth (Fig. 6A). Next, we developed MP xenografts and treated tumor-bearing mice with trametinib (1 mg/kg/day) or vehicle control. Consistent with the in vitro data, trametinib successfully suppressed MP cell growth in vivo (Fig. 6B and Table S4). Mechanistically, tumors from xenografted MP cells showed significant activation of the MAPK signaling, which was significantly decreased/abolished, as assessed by reduced immunoreactivity for phosphorylated/activated ERK proteins in trametinib-treated samples (Fig. 6C). In addition, Ki67 and cleaved-caspase 3 (C-C3) staining indicated that trametinib treatment decreases proliferation and induces apoptosis in c-MET/sgp53 HCC cell xenografts (Fig. 7). Subsequently, we treated MP cells with cabozantinib and trametinib, either alone or in combination. Intriguingly, concomitant treatment of MP cells with cabozantinib and trametinib induced more potent growth inhibition, suggesting a potential synergistic effect of the combination therapy (Fig. S13).

Altogether, these data underline the therapeutic potential of MEK/ERK inhibitors for treating p53-deficient HCC.

## Discussion

Somatic mutations of the p53 tumor suppressor gene occur in ~50% of overall human tumors and represent one of the most common genetic variations of HCCs [25, 26]. In most cases, *TP53* mutations abolish the functions of the p53 protein, such as gene transcription, DNA synthesis and repair, cell cycle arrest, senescence, and apoptosis [26, 27]. It has been shown that such inactivation mutations could lead to HCC onset and tumor progression [28–30]. Therefore, stabilizers of WT p53 or drugs that revert mutant p53 back to WT function have long been investigated for HCC treatment. However, the preclinical HCC models for studying p53 are still limited. According to the *TP53* database (https://tp53.isb-cgc.org/), only a few murine liver cancer models with engineered p53 are reported in the scientific literature (Table S5). In addition, most of the mouse models were developed as transgenic mouse strains. However, it takes long latency periods for these transgenic mouse strains to develop liver tumors, which significantly constrains their availability. In the current study, we generated a murine HCC model with hydrodynamic transfection of c-MET oncogene and CRISPR-Cas9 mediated KO of p53, which could be efficiently applied to study *TP53* null HCC development in vivo.

To further delineate the mechanisms associated with *TP53* inactivation-related hepatocarcinogenesis, we have established malignant murine cell lines from this c-MET/sgp53-driven murine HCC model. Notably, these cells express high levels of p-ERK1/2 compared to c-MYC tumor-derived cell lines (HCC3 and HCC4) (Fig. S14). Significantly, the MP cells can be effectively implanted into immunocompetent mice with resultant subcutaneous tumor formation. Using RNA-Seq on these cells, we identified a cluster of genes upregulated in c-MET/sgp53 tumors. Comparing these genes with those altered in p53-mutated tumors without c-MET activation will help to determine whether such genes are specific to this combination of genetic alterations. However, the deletion of *p53* could not induce HCC per se [19], making it impossible to perform such a comparison. One possible strategy might be comparing those genes with DEGs in other *p53* null tumors, for example, the c-MYC/sgp53 HCCs [31]. These analyses will assist us in identifying genes or pathways which might be specific to the loss of *TP53* in HCC. Thus, the MP cells could be applied to establish orthotopic murine HCC models. In addition, this model could have significant utility in investigating oncogenic signaling pathways in HCCs as it allows the manipulation of the murine cells before transplantation. For instance, transfection of the cells with inducible genes or inhibition constructs may elucidate the impact of various oncogenes in HCC progression and survival. Moreover, this model can be exploited to examine the immunologic response and stroma formation in HCC and investigate new therapies for HCC.

For p53 mutated HCCs, the current drug development strategies include restoring wild-type p53 conformation and transcriptional activity, inducing the degradation of mutated p53, and inhibiting the interaction of p53 and its negative regulatory factor MDM2. However, the heterogeneity of p53 mutations in tumors limits the use of these drugs. Thus, synthetic lethal effects can be a promising therapy for a wide range of functional p53 mutations in HCC. Here, our murine HCC model and the related HCC cell line with deletion of p53 could be a valuable preclinical model in exploring targeted treatments for p53-loss-of-function HCCs in vitro and in vivo.

The current study reveals that cabozantinib and trametinib inhibit the growth of c-MET/sgp53 HCC xenografts. These findings highlight the importance of biomarker-based targeted therapies for effective cancer treatment. In phase I clinical study, trametinib, and sorafenib were used to treat unselected patients with hepatocellular carcinoma. Unfortunately, the resulting clinical data indicated this combination therapy had limited efficacy in advanced HCC [32]. However, this is likely because trametinib may be effective in HCCs harboring *TP53* null mutations. Therefore, it is critical to identify a potential biomarker(s) of response to the effective therapy for HCCs. Thus, preclinical and clinical studies are required to examine the therapeutic efficacy of trametinib against *TP53* null human HCCs.

Our study has several limitations. The trametinib sensitivity data are based on three HCC cell lines (Hep3B, LM9, and HLE) and one hepatoblastoma cell line (HepG2). They are not isogenic models. Unfortunately, there is no commercially available HCC cell line with wild-type *TP53*. Clearly, there is a need to develop human HCC cell lines with wild-type *TP53*, and these cell lines would be critical to validate the specificity of trametinib against *TP53* null HCC cells. The c-MET signature of these cell lines has been previously analyzed [20], revealing that Hep3B cells have low c-MET activation. The fact that Hep3B cells have low c-MET activity suggests that the sensitivity towards trametinib is likely independent of c-MET activation status.

It is important to note that HCC is a heterogeneous disease with many subtypes. Therefore, identifying reliable biomarkers for patient selection is critical for precision HCC treatment. As revealed in our previous work [33], c-MET high signature in combination with PTEN loss of function mutation also predicted poor prognosis of HCC patients and showed sensitivity to FASN inhibitors rather than tyrosine kinase inhibitors. Here, we provide new data suggesting that c-MET high/*TP53* null tumors represent an HCC subset that trametinib could specifically target. Based on the TCGA-LIHC dataset, 23 human HCC samples displayed c-MET activation and *TP53* null mutation concomitantly, representing 6% of the human HCC collection. Each year, approximately 900,000 new cases of liver cancer occur worldwide [1]. Therefore, roughly 50,000 HCCs display *TP53* null mutation and c-MET amplification. These patients may benefit from the targeted treatment identified in this study.

## Materials and methods

### Plasmids and reagents

The plasmids used in this study, including pT3-EF1α-c-MET (human c-MET or hMET) and pCMV-sleeping beauty transposase (SB), have been described in our previous studies [13, 17, 34]. pX330-sgp53 plasmid was obtained from Addgene (#59910). Plasmids were purified using the Endotoxin-free Maxi prep kit (Sigma-Aldrich, St. Louis, MO, USA). Cabozantinib and trametinib were purchased from LC Laboratories (Woburn, MA, USA). Niclosamide and metformin were obtained from Sigma-Aldrich (St. Louis, MO, USA).

### Hydrodynamic tail vein injection

FVB/N mice were obtained from Charles River Laboratories (Wilmington, MA, USA). To generate the c-Met/sgp53 HCC model, 20 μg pT3-EF1α-c-MET and 20/40 μg pX330-sgp53 along with 0.8 μg pCMV/SB plasmids in 2 ml saline (0.9% NaCl) were delivered to 6–8 week-old mice (half male and half female) by hydrodynamic tail vein injection. Mice were housed, fed, and monitored following protocols approved by the Committee for Animal Research at the University of California, San Francisco, and the University of Hawaii Cancer Center.

### Generating the stably passaged cell line from c-MET/sgp53 HCC

Mice were humanely euthanized and rinsed in 70% ethanol. The liver tumor (2–3 g) was dissected, washed in PBS, and minced into ~1 mm fragments using a scalpel blade. All procedures were operated in the hood using autoclaved dissecting tools. The tumor fragments were digested with 0.6 mg/L collagenase (Sigma-Aldrich, C5138) at 37 °C for 15–30 min, then filtered through 100 μm nylon mesh cell strainer (BD, 352360) and spun (700–1000 rpm, 5 min). The cell pellet was resuspended and cultured in 5 ml DMEM with 10% fetal bovine serum (FBS) and 1% Penicillin/Streptomycin at 37 °C and 5 % CO_2_ for ~4 weeks. The primary liver tumor cells were generated. To obtain a stably passaged cell line, 1 × 10^7^ primary tumor cells were injected into flanks of 6–8 week-old FVB/N mice. When the subcutaneous tumor developed, tumor tissue was dissected into small pieces and digested with trypsin (Sigma-Aldrich, T4049). The new generation of liver tumor cells (P0) were cultured and passaged in vitro for 3 times. Then 1 × 10^7^ “P0” cells were implanted into the flanks of a new recipient FVB/N mouse. These procedures were repeated for three rounds. The stably passaged cell line from c-MET/sgp53 HCC (MP cell) was finally generated.

### Mice xenografts and treatment

To develop mice xenografts, 200 μl (1 × 10^7^ MP cells) suspension of tumor cells in Matrigel (Corning, 354248) were inoculated subcutaneously into the right flank of 6 ~ 8 week-old FVB/N mice (females). On day 14 post-implantation, when the tumor size reached about 100–150 mm^3^, mice were randomized into 5 groups for treatment. Metformin (250 mg/kg/day), niclosamide (100 mg/kg/day), cabozantinib (60 mg/kg/day), trametinib (1 mg/kg/day), or vehicle was orally administered daily via gavage. Tumor volume was measured every three days and estimated from the caliper using the formula *V* = *A* × *B*^2^/2 (*A* is the largest diameter, *B* is the smallest diameter). In addition, body weight was measured every day. After 3 weeks of treatment, all mice were sacrificed, and tumors were harvested for further analysis.

### HCC cell lines

The following human HCC cell lines were used in this study: LM9, HLE, HepG2, and Hep3B. They were obtained from the American Type Culture Collection (ATCC, Manassas, VA, USA). In addition, Dr. Felsher of Stanford University kindly provided the HCC3-4 and HCC4-4 cell lines, which were isolated from c-Myc mouse liver tumors. All cell lines were grown in Dulbecco’s Modified Eagle Medium (DMEM) supplemented with 10% FBS and penicillin/streptomycin (Gibco, Grand Island, NY, USA) in a 5% CO_2_ atmosphere at 37 °C. For IC50 determination, cells were seeded in 24-well plates and treated with a gradient concentration of trametinib in triplicate for 48 hours. Then cells were enumerated by crystal violet staining. After washing, stained cells were incubated in lysis solution and shaken gently on a rocking shaker for 10 min. Diluted lysate solutions were added to 96-well plates, and the OD value was measured at 590 nm with the BioTek ELX808 Absorbance Microplate Reader (ThermoFisher Scientific, MA, USA). The IC50 values were calculated using the Prism 9.0 software (GraphPad Software Inc.). All experiments were repeated at least three times.

### Histology and immunohistochemistry

Mouse liver tissues and tumor tissues were fixed in 4% paraformaldehyde, embedded in paraffin, and sectioned at 5 μm. Then, hematoxylin and eosin (H&E), and IHC staining were performed. For antigen retrieval, de-paraffinized slides were incubated in antigen retrieval buffer (10 mM sodium citrate buffer, pH 6.0) and microwaved for 10 min. After a blocking step with the 5% goat serum and Avidin-Biotin blocking kit (Vector Laboratories Inc., Burlingame, CA), the sections were incubated with the primary antibodies overnight at 4 °C. To quench the endogenous peroxidase, slides were then subjected to 3% hydrogen peroxide for 10 min, and subsequently, the secondary antibody was applied for 30 min at room temperature. The immunoreactivity was visualized with the Vectastain ABC Elite kit (Vector Laboratories Inc.) and DAB (Vector Laboratories, Inc.). Slides were counterstained with hematoxylin. The following primary antibodies were used: anti-p53 (sc-126; Santa Cruz, CA, USA), anti-CK19 (ab52625; Abcam, Cambridge, United Kingdom), anti-HNF4a (ab18604; Abcam), anti-Ki67 (MA5-14520; Thermo Fisher Scientific), anti-p-ERK (4370; Cell Signaling Technology, Danvers, MA, USA), and anti-Cleaved-Caspase 3 (9664; Cell Signaling Technology) were used in the present investigation.

### Protein extraction and Western blot analysis

Frozen liver tumors were homogenized in Mammalian Protein Extraction Reagent (Thermo Fisher Scientific) containing the Complete Protease Inhibitor Cocktail (Thermo Fisher Scientific). Protein concentrations were determined with the Bio-Rad Protein Assay Kit (Bio-Rad, Hercules, CA, USA). The Supernatant was denatured by boiling in 2× Laemmli sample buffer (1610737, Bio-Rad). Equal loading was assessed by probing the membranes with the GAPDH or β-actin antibody. Aliquots of 30 μg protein lysates were separated by SDS-PAGE (M00654, GenScript, Piscataway, NJ, USA) and transferred onto PVDF membranes (Bio-Rad). Membranes were blocked in 10% non-fat milk in Tris-buffered saline containing 0.05% Tween-20 and incubated with primary antibodies at 4 °C overnight. Then membranes were incubated with horseradish peroxidase-secondary antibody (Jackson ImmunoResearch Laboratories Inc., West Grove, PA, USA) for 1 h at room temperature and developed with ClarityTM Western ECL Substrate (170-5061, Bio-Rad). The antibodies used are as follows: anti-p53 (sc-126; Santa Cruz Biotechnology), anti-c-MET (71–8000; Thermo Fisher Scientific), anti-p-MET^Tyr1234/1235^ (3077, Cell Signaling Technology), anti-ERK1/2 (9102; Cell Signaling Technology) and anti-p-ERK1/2^Thr202/Tyr204^ (4370; Cell Signaling Technology), anti-GAPDH (5174; Cell Signaling Technology), and anti-β-actin (4970; Cell Signaling Technology).

### Mouse genomic DNA extraction and sequencing

According to the manufacturer’s instructions, mouse genomic DNA was extracted from frozen mouse tissue samples using the Mouse Direct PCR Kit (Bimake, Houston, TX, USA). The amplification conditions were 94 °C for 5 min, followed by 35 cycles of 94 °C for 20 s, 50 °C for 30 s, and 72 °C for 30 s. The sequences of the primers are as follows: Forward: CCTACTGGATGTCCCACCTTCT; Reverse: CAGACACCCAACACCATACCA. For individual clonal sequencing, PCR products were purified and inserted in the pGEM®-T Easy Vector (Promega, Madison, WI, USA) according to the manufacturer’s instructions. Next, clones were cultured, and plasmids were extracted using the Zyppy Plasmid Miniprep kit (Genesee Scientific, San Diego, CA, USA). The inserted sequence was subsequently sequenced using T7 primers.

### RNAseq analysis

Total RNA was extracted from mouse sgp53/c-MET HCCs (*n* = 3), MP cells (*n* = 3), and FVB/N normal livers (*n* = 6) using Quick-RNA Miniprep Kit (Zymo Research, Irvine, CA, USA). For RNA extraction, the c-MET/sgp53 HCCs were collected from male mice at 17, 20, and 22 weeks post-injection. The normal livers were collected from FVB/N male mice aged 21–28 weeks. The normal liver tissues were collected from wild-type FVB mice. The RNA quality control was determined using Agilent RNA 6000 Nano Kit (Agilent Technologies, CA, USA) and Bioanalyzer (Agilent Technologies, CA, UAS). Library preparation and sequencing were performed by Novogene (Sacramento, CA, USA). Reads quality was controlled by fastqc (v0.11.7). Gene read counts were in Ensembl Gene ID and converted to Entrez Gene ID using Bioconductor Package Maintainer org.Mm.eg.db. Not annotated, duplicate Entrez IDs and genes without symbols were removed. R package “edgeR” were used to identify the DEGs. DEGs were limited by a *p*-value of 0.05 and an FDR (False Discovery Rate) of 0.05. The Venn diagram was drawn using the VennDiagram package. Kyoto Encyclopedia of Genes and Genomes (KEGG) pathway enrichment analysis were performed using ggplot2 package. The RNAseq data for this study were deposited in the Gene Expression Omnibus database (GSE229764).

### Human data HCC TCGA retrieval and analysis

To investigate the relationship of c-MET activation with *TP53* mutation status in human HCC samples, TGCA data sets were retrieved based on the cBioPortal for Cancer Genomics (http://www.cbioportal.org). The overall sample size is 410, including 50 surrounding liver tissues (ST) and 360 HCC samples with *TP53* mutation data. RNA sequencing data were analyzed in R using multiple packages. The analysis of c-MET activation status was performed as previously described [17]. In brief, we extracted genes from the “KAPOSI_LIVER_CANCER_MET_UP” gene set, which contains 18 genes that were upregulated in liver cancer samples in response to c-MET activation. Heatmap was generated using the Complex heatmap [35]. The data were standardized with mean as “0” and standard deviation (SD) as “1” and ordered by ascending average of 18-gene expression of each sample from left to right. As the weight of each gene is 1 in this gene set, samples with averages more than the average plus 1.5-fold SD of the ST group were considered as HCC with “c-MET activation”. The follow-up survival data were obtained using the R package TCGAbiolinks.

### Statistical analysis

The Prism 9.0 software (GraphPad Software Inc.) was used to analyze the data. Statistical analysis was performed using Student’s *t*-test, Mann–Whitney test, Welch’s *t*-test, and Log-rank (Mantel-Cox) test analyses. The data were expressed as the mean ± SD (*P < 0.05; **P < 0.01; ***P < 0.001) of at least three independent experiments.

## Supplementary information

Supplementary Figures

Supplementary Table S1

Supplementary Table S2

Supplementary Table S3

Supplementary Table S4

Supplementary Table S5

Supplementary Table S6

Original western blots

checklist

## Data Availability

The datasets generated and analyzed during the current study are available from the corresponding author upon reasonable request.
