# Supplementary material for: Loss of TP53 cooperates with c-MET overexpression to drive hepatocarcinogenesis
Source: Cell Death Dis. 2023 Jul 27;14(7):476. doi: 10.1038/s41419-023-05958-y (PMC10374654; doi:10.1038/s41419-023-05958-y)
Supplement: Supplementary file 1 — Supplementary Figures [file 41419_2023_5958_MOESM1_ESM.docx]

**Supplementary Figures**


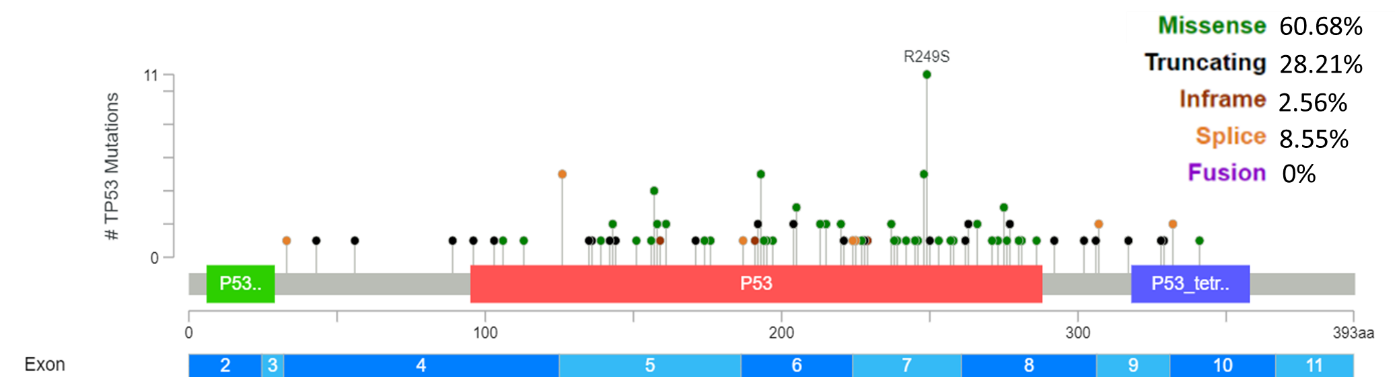
**Supplementary Figure 1. The summarizing scheme for the TP53 mutations in human HCC samples base on TCGA dataset.**


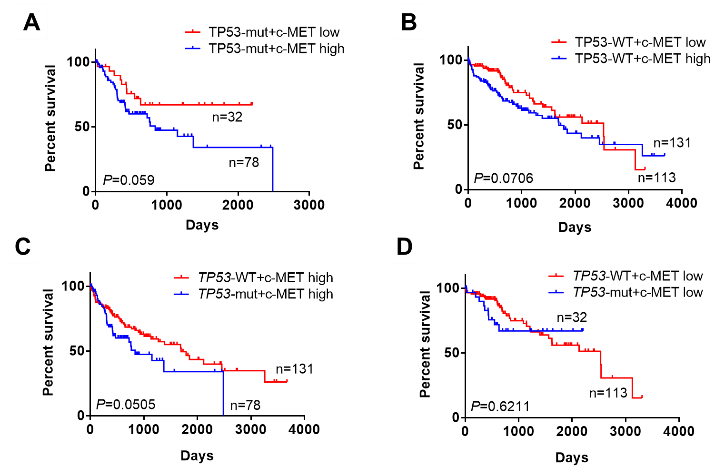
**Supplementary Figure 2. Survival curves of patients in other categories.**

Survival curves of patients in other categories including concomitant (A) TP53 mutant/c-MET low vs. TP53 mutant/c-MET high, (B) TP53 WT/c-MET low vs. TP53 WT/c-MET high, (C) TP53 mutant/c-MET high vs. TP53 WT/c-MET high, and (D) TP53 mutant/c-MET low vs. TP53 WT/c-MET low. Log-rank (Mantel-Cox) test were applied.

**Supplementary Figure 3. The mRNA expression of HCC-related genes (*Afp*, *Gpc3* and *Prom1*) and cell proliferation-associated genes (*Ccnb1*, *Ccne1*, *Cdk6*, *Bub1* and *Mki67*) in normal liver and c-MET/sgp53 tumors.** At least 3 mice in each group were assayed. Data are shown as mean ± SD. Welch’s t test: ***P* < 0.01, ****P* < 0.001, *****P* < 0.0001.


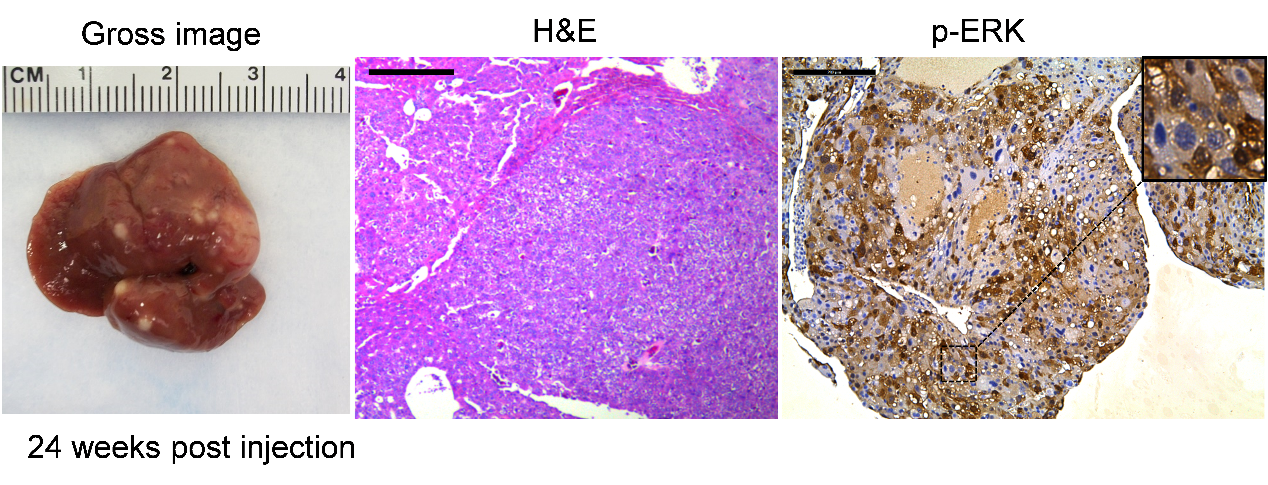


**Supplementary Figure 4. Representative gross images, H&E staining, and p-ERK staining in tumor lesions from a c-MET/sgp53 mouse liver.** Plasmids injection dose: 20μg pT3-EF1α-c-MET, 20μg pX330-sgp53 and 0.8μg pCMV/SB; Scale bar = 200 μm.


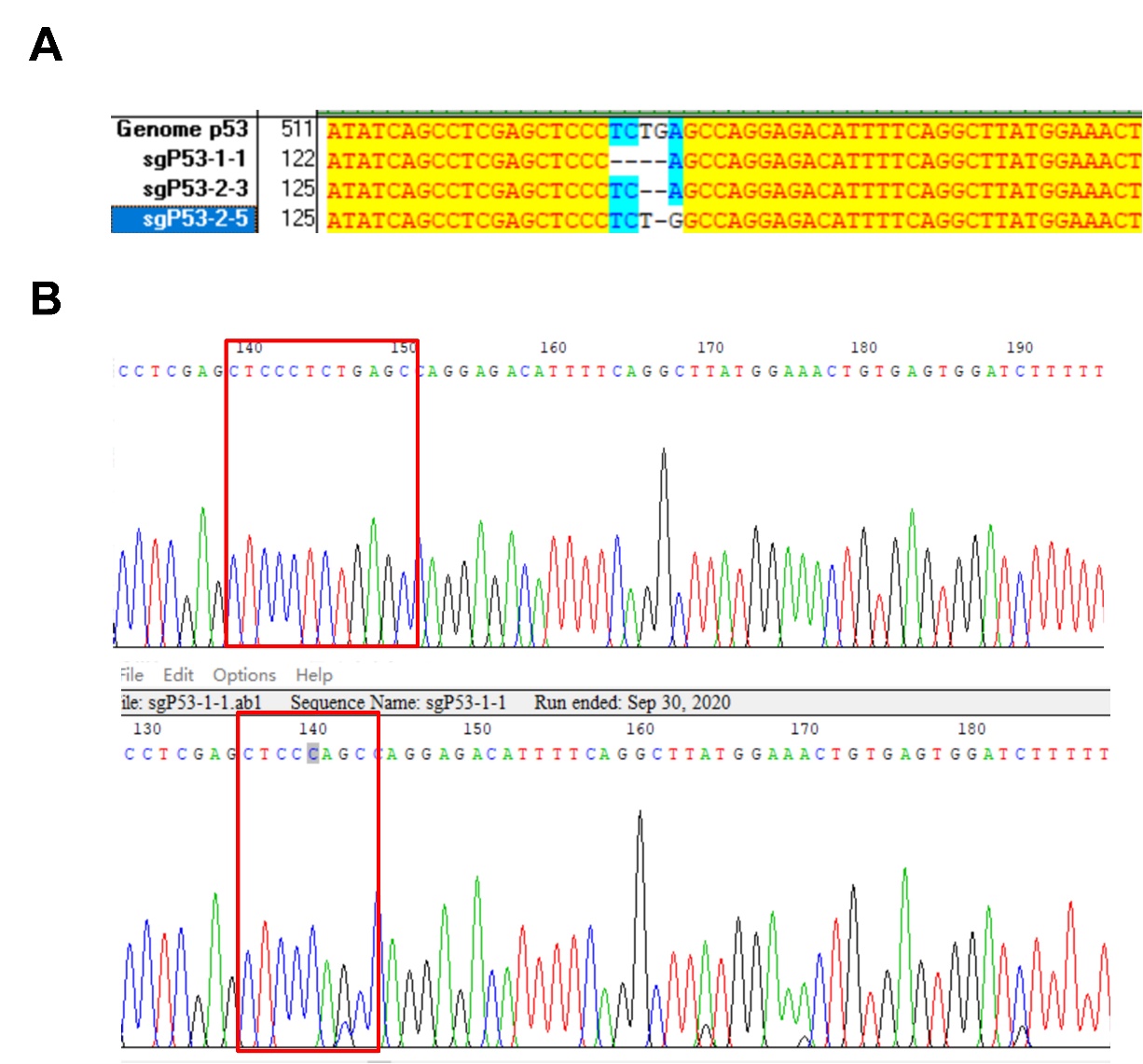


**Supplementary Figure 5. Genomic sequencing and validation of *Trp53* deletion in mouse HCCs.** (A) The Sanger sequencing results from different clones with sgp53 induced mutation from mouse tumor nodules, confirming the nucleotide deletions of *Trp53* on its genomic locus. (B) Original wild-type mouse *Trp53* alleles and one of the representative nucleotide deletions of *Trp53* caused by sgp53.


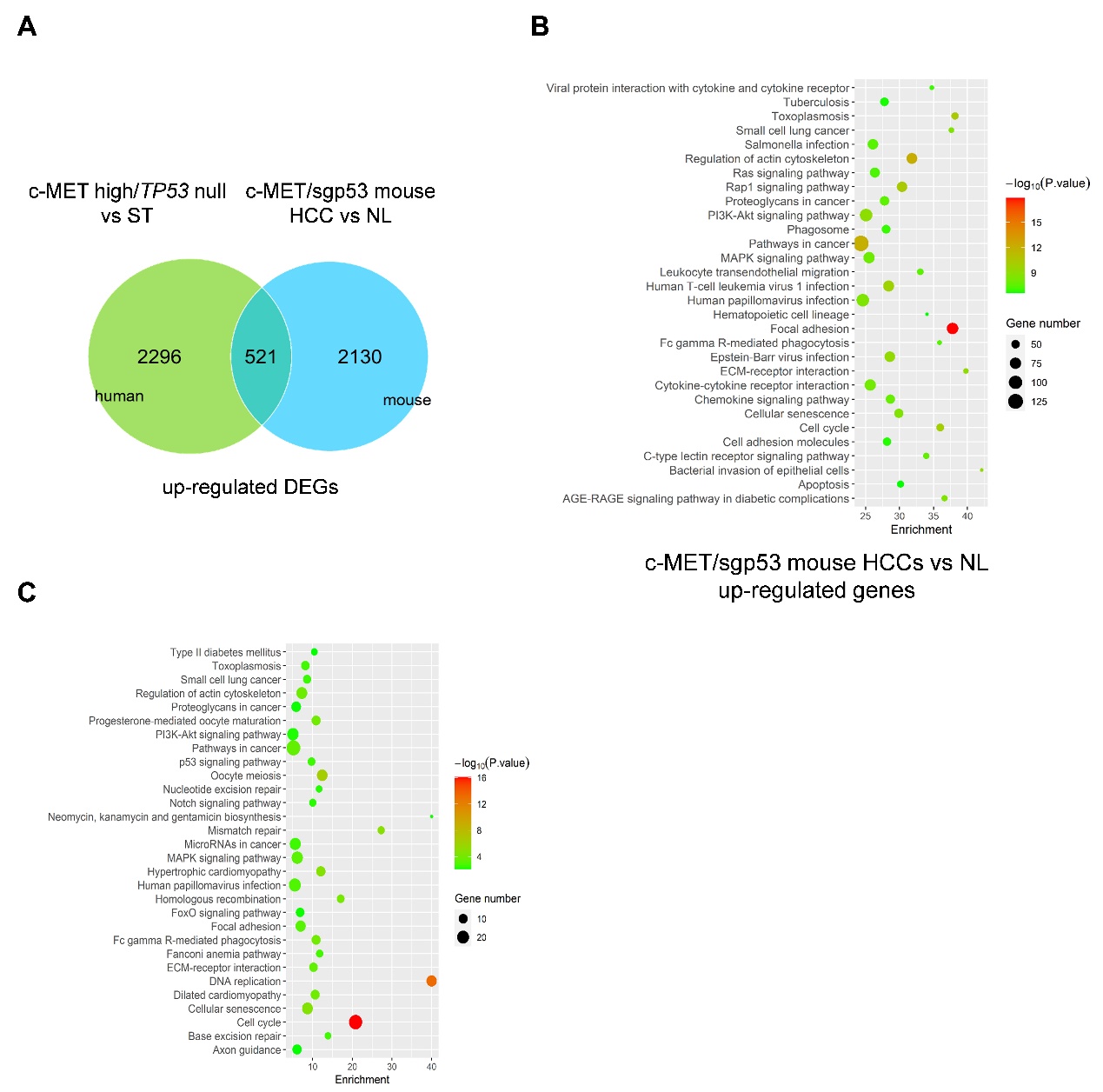


**Supplementary Figure 6. The details of KEGG pathway analysis of up-regulated DEGs in the c-MET/sgp53 HCCs.** (A) Numbers of overlapping up-regulated DEGs in c-MET/sgp53 HCC and c-MET-high/*TP53*-null human HCC. (B) KEGG analysis of up-regulated genes in the c-MET/sgp53 HCCs as compared to normal livers. (C) KEGG analysis of overlapping up-regulated DEGs in c-MET/sgp53 HCCs and c-MET-high/*TP53*-null human HCCs. NL, normal livers; ST, surrounding tissues; DEG, differentially expressed genes. (This figure is related to Figure 3B and C).


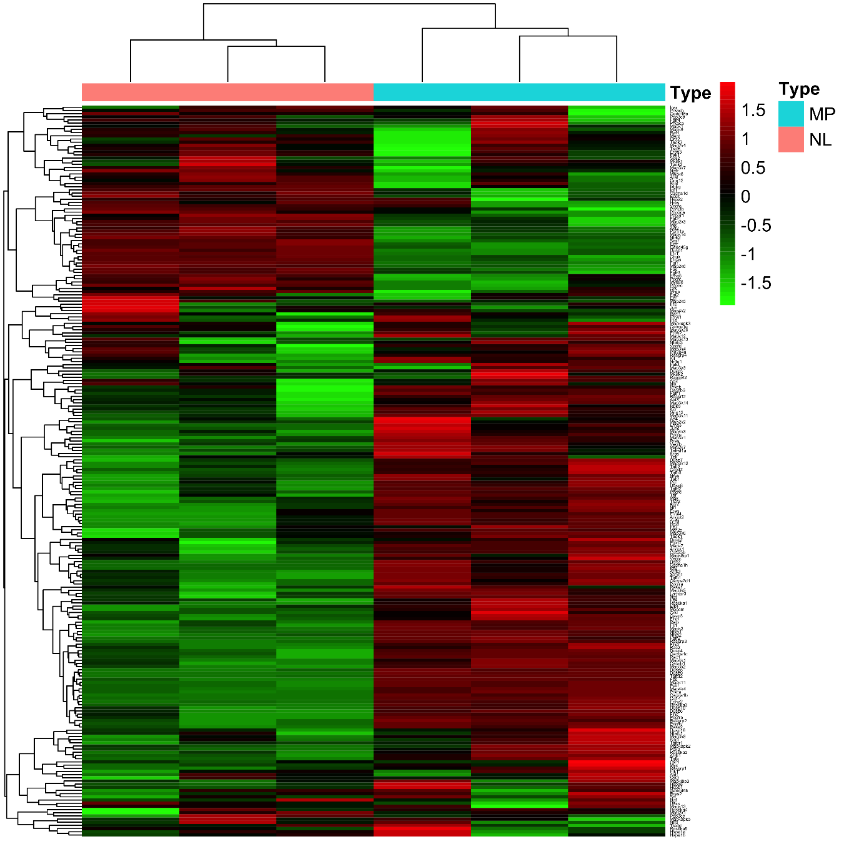


**Supplementary Figure 7. Heatmap of MAPK pathway genes in c-MET/sgp53 mouse HCC vs normal liver.**


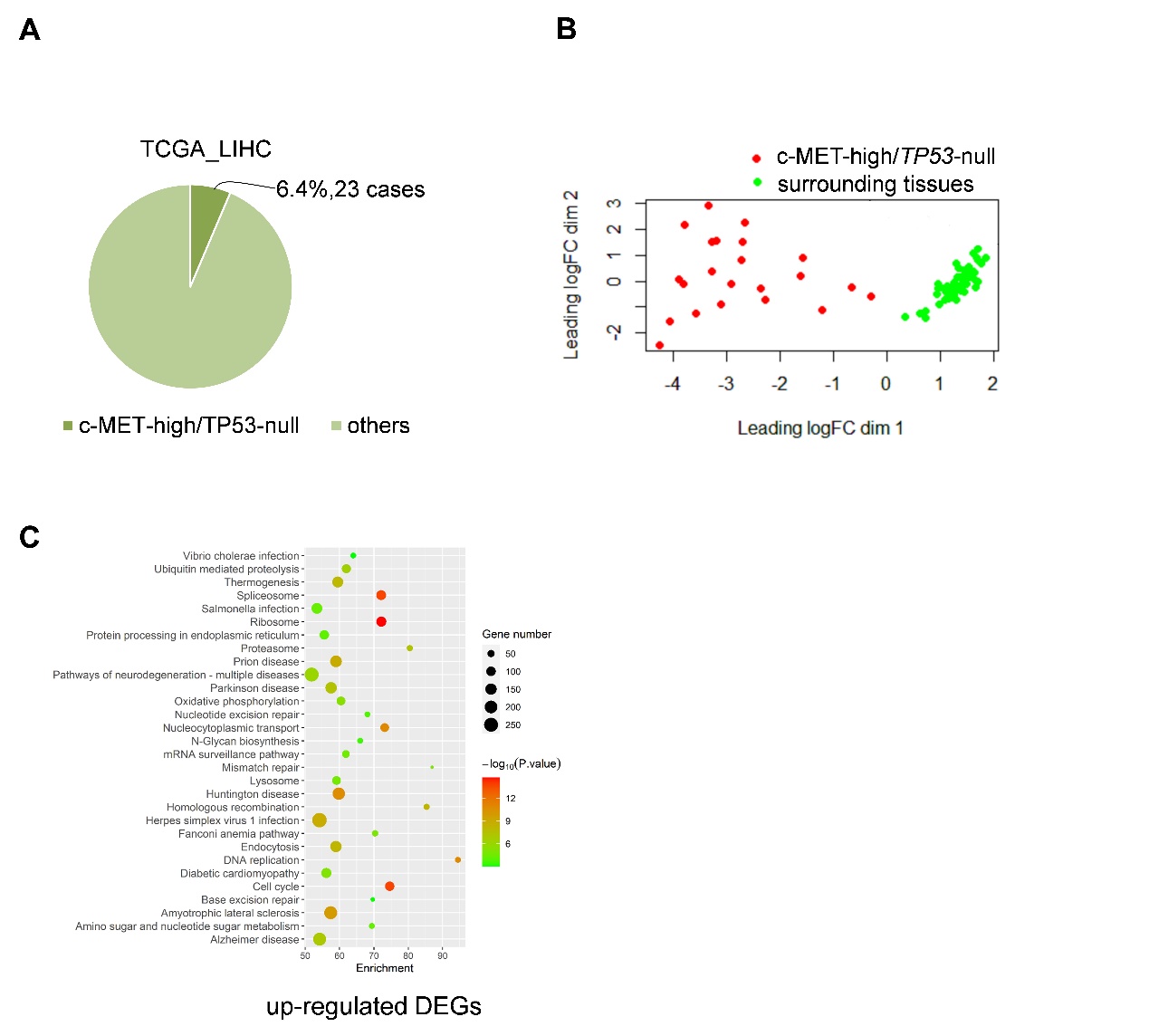


**Supplementary Figure 8. A subset of human HCC samples harboring concomitant c-MET activation and *TP53* deletion based on the TCGA-LIHC dataset.**

(A) The number and percentage of c-MET-high/*TP53*-null human HCC cases in TCGA-LIHC database. (B) Genetic dissimilarity among the samples in c-MET-high/*TP53*-null human HCCs and surrounding tissues as demonstrated by multidimensional scaling (MDS). (C) KEGG analysis of up-regulated genes in c-MET-high/*TP53*-null human HCCs as compared to surrounding tissues.


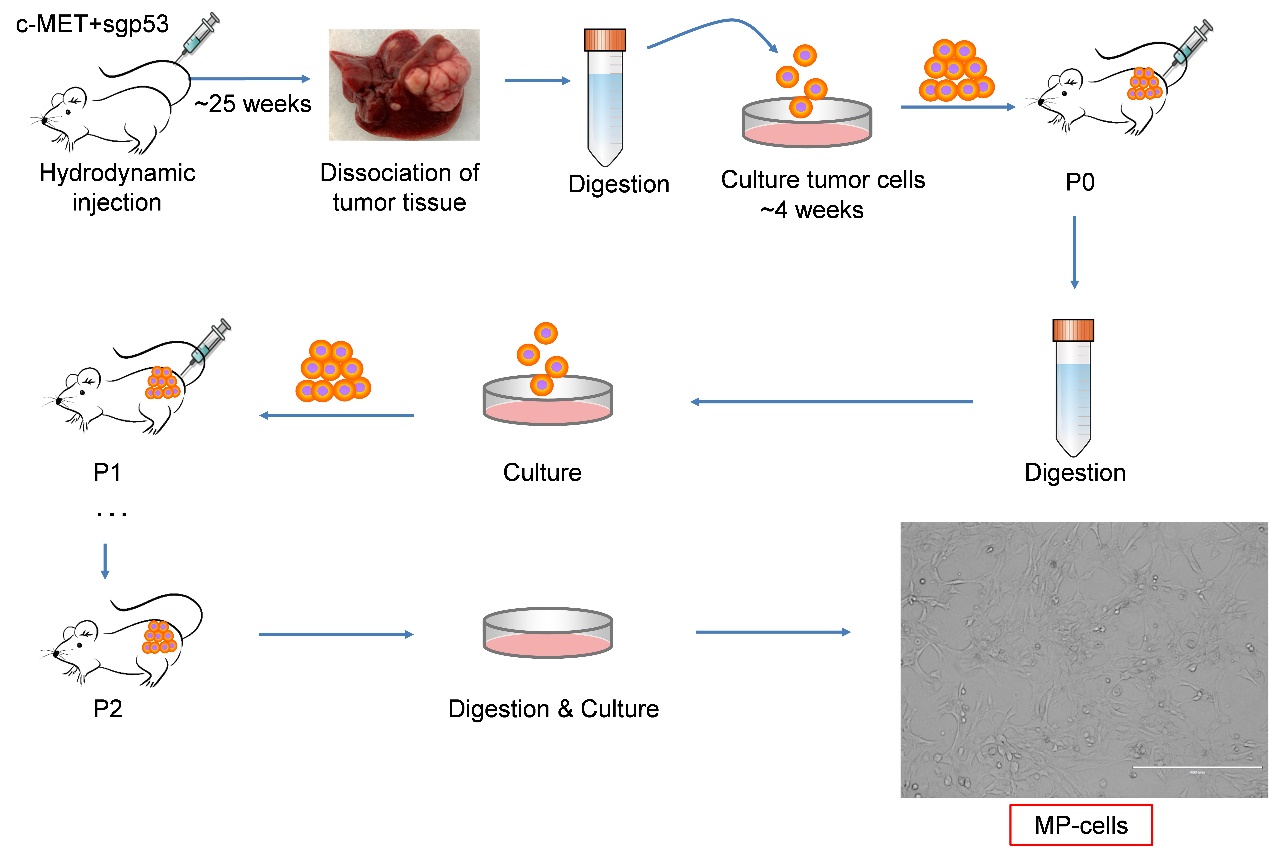


**Supplementary Figure 9. Illustration of generating the stably passaged cell line from c-MET/sgp53 HCC.**

**
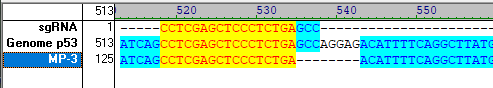
**

**Supplementary Figure 10. Genomic sequencing and validation of *Trp53* deletion in MP cells.**


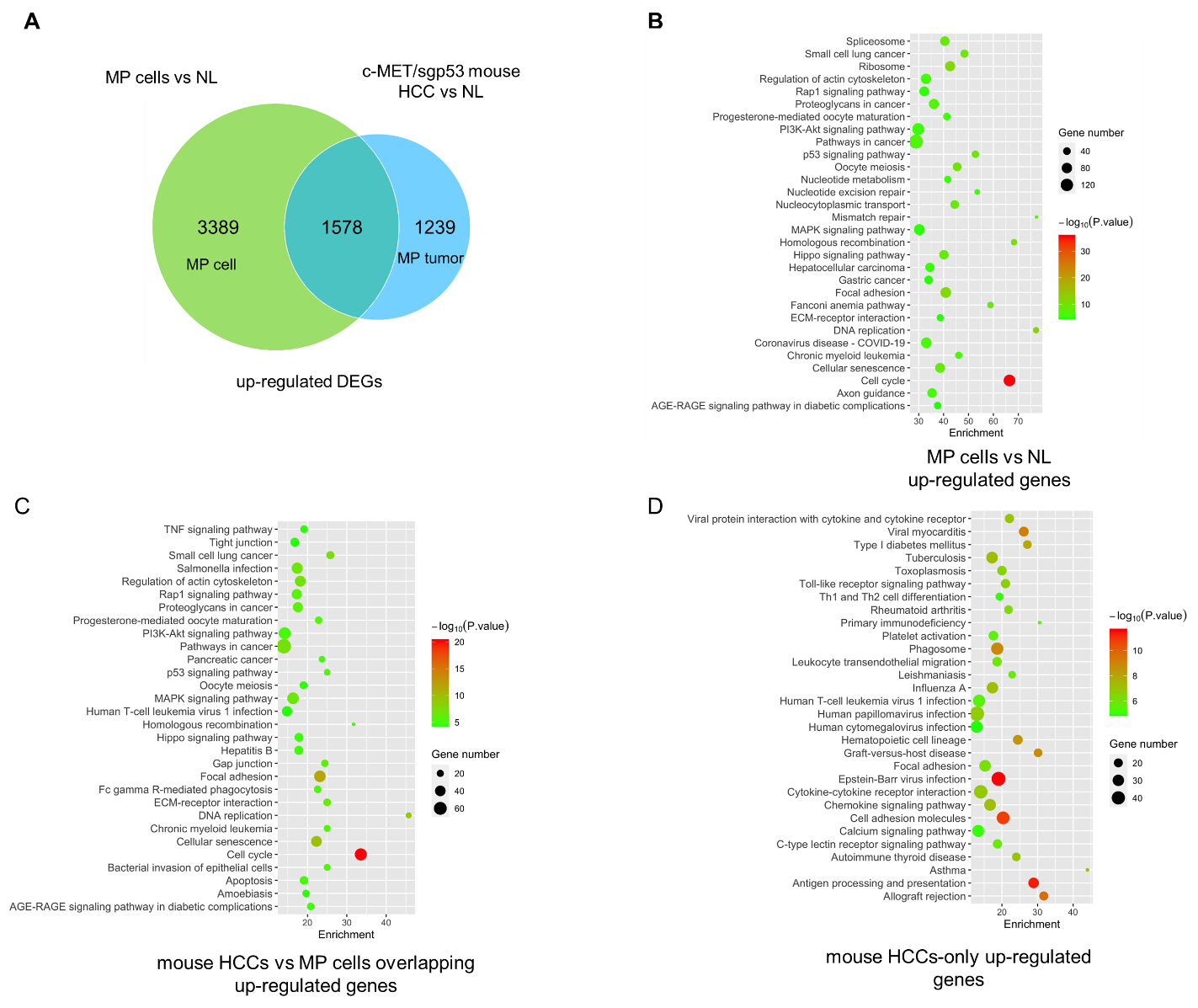


**Supplementary Figure 11. RNA sequencing data show the similar pattern in MP cells with c-MET/sgp53 HCCs.**

(A) Numbers of overlapping up-regulated DEGs in MP cells and c-MET/sgp53 HCCs. KEGG analysis of (B) up-regulated genes in MP cells as compared to normal livers; (C) overlapping up-regulated DEGs in c-MET/sgp53 HCCs and MP cells. (D) DEGs only up-regulated in MP tumors as compared to MP cells. NL, normal livers; ST, surrounding tissues; DEG, differentially expressed genes.

**Supplementary Figure 12. M EK inhibitors show a higher inhibition score in *TP53*-null human HCC cell line.**

The IC50 value of *TP53*-null HCC cell line Hep3B (*TP53*-null) and other human HCC cell lines (*TP53*-mut or WT) for MEK inhibitors (SCH772984, ERK2440, ERK6604 and trametinib) base on the Genomics of Drug Sensitivity in Cancer database (www.cancerRxgene.org). IC50, half maximal inhibitory concentration.
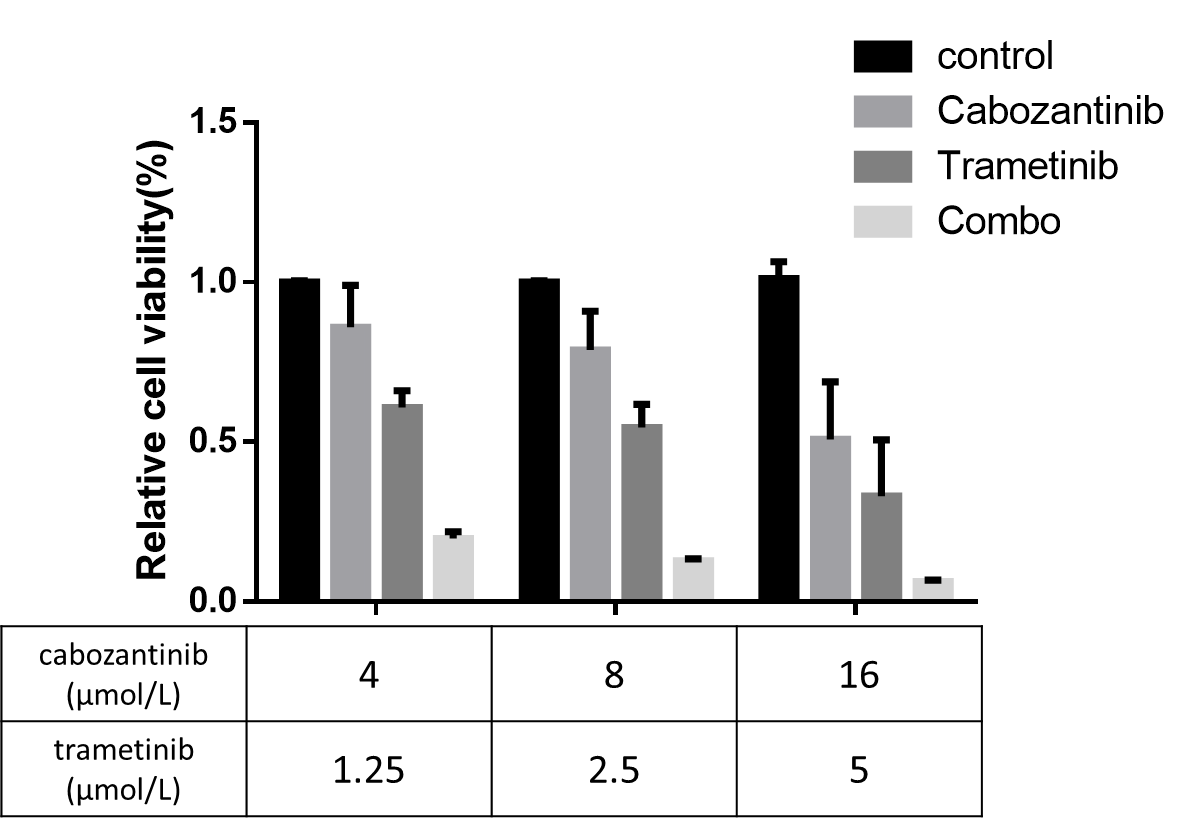
**Supplementary Figure 13. Trametinib and cabozantinib showed synergistic effect in the c-MET/sgp53 HCC cells**


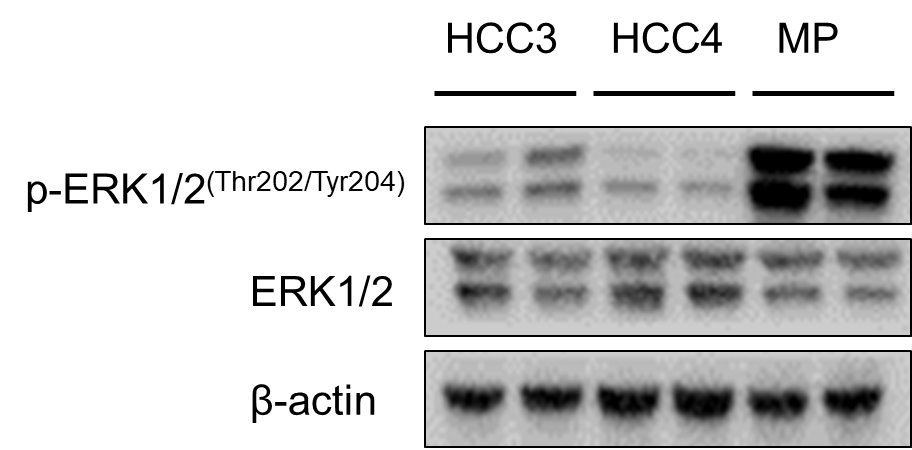


**Supplementary Figure 14. Western blotting analysis of ERK signaling in MP, HCC3 and HCC4 mouse HCC cells.**
