# Supplementary material for: Loss of TP53 cooperates with c-MET overexpression to drive hepatocarcinogenesis
Source: Cell Death Dis. 2023 Jul 27;14(7):476. doi: 10.1038/s41419-023-05958-y (PMC10374654; doi:10.1038/s41419-023-05958-y)
Supplement: Supplementary file 3 — Supplementary Table S2 [file 41419_2023_5958_MOESM3_ESM.docx]

**Supplementary Table S2. List of mice used for hydrodynamic injection.**

| **Mice strain** | **Plasmid injected (μg)** | **Weeks post injection** | **Body weight (g)** | **Liver weight (g)** |
| --- | --- | --- | --- | --- |
| FVB/N | c-Met (20) + sgp53 (20) + SB (0.8) | 24 | 32.3 | 5.4 |
|  |  | 28.7 | 37 | 6.9 |
|  |  | 28.7 | 33 | 6.9 |
|  |  | 20 | 36 | 6.3 |
|  |  | 24.5 | 40.4 | 3.3 |
|  |  | 25 | 34.6 | 6.2 |
|  |  | 25 | 33.2 | 4.4 |
|  |  | 26 | 34.6 | 3.4 |
|  | c-Met (20) + sgp53 (40) + SB (0.8) | 17 | 34.5 | 3.6 |
|  |  | 17 | 36.6 | 9.9 |
|  |  | 22.1 | 36.2 | 5.7 |
|  |  | 28 | 35.5 | 3.6 |
|  |  | 32 | 45.6 | 3.8 |
|  |  | 32 | 38.2 | 7.7 |
