# Supplementary material for: Loss of TP53 cooperates with c-MET overexpression to drive hepatocarcinogenesis
Source: Cell Death Dis. 2023 Jul 27;14(7):476. doi: 10.1038/s41419-023-05958-y (PMC10374654; doi:10.1038/s41419-023-05958-y)
Supplement: Supplementary file 5 — Supplementary Table S4 [file 41419_2023_5958_MOESM5_ESM.docx]

**Supplementary Table S4. Detailed information for the xenografts experiment.**

| **Mice strain** | **Treatment group** | **A**  **(mm)** | **B**  **(mm)** | | | **Tumor volume (mm^3^)** | **Tumor weight (g)** |
| --- | --- | --- | --- | --- | --- | --- | --- |
| FVB/N | Vehicle | 16.98 | 10.4 | | | 918.28 | 1.55 |
|  |  | 12.54 | 11.33 | | | 804.87 | 0.93 |
|  |  | 17.93 | 12.7 | | | 1464.24 | 1.56 |
|  |  | 14.28 | 10.75 | | | 825.12 | 1.32 |
|  | Cabozantinib | 6.51 | 4.33 | | | 61.03 | 0.07 |
|  |  | 4.02 | 2.87 | | | 16.56 | 0.02 |
|  |  | 4.51 | 3.46 | | | 27.00 | 0.03 |
|  |  | 6.11 | 4.41 | | | 59.41 | 0.08 |
|  | Metformin | 13.68 | 11.93 | | | 973.50 | 1.32 |
|  |  | 17.12 | 10.92 | | | 1020.75 | 1.56 |
|  |  | 17.55 | 13.51 | | | 1601.61 | 1.66 |
|  | Niclosamide | 14.61 | 9.55 | | | 666.23 | 0.66 |
|  |  | 16.98 | 10.8 | | | 990.27 | 1.06 |
|  |  | 14.39 | 10.05 | | | 726.71 | 0.76 |
|  |  | 14.58 | 10.88 | | | 862.95 | 0.93 |
|  | Trametinib | 8.68 | 7.5 | | | 244.13 | 0.29 |
|  |  | 6.26 | 5.21 | | | 84.96 | 0.06 |
|  |  | 8.23 | 5.08 | | | 106.19 | 0.12 |
|  |  | 9.14 | 6.73 | | | 206.99 | 0.19 |
| A is the largest diameter, B is smallest diameter. | | |  |  |  |  |  |
