# Supplementary material for: Loss of TP53 cooperates with c-MET overexpression to drive hepatocarcinogenesis
Source: Cell Death Dis. 2023 Jul 27;14(7):476. doi: 10.1038/s41419-023-05958-y (PMC10374654; doi:10.1038/s41419-023-05958-y)
Supplement: Supplementary file 6 — Supplementary Table S5 [file 41419_2023_5958_MOESM6_ESM.docx]

| **Allele designation** | **Model description** | **Mouse strains** | **Latency** | **Reference** |
| --- | --- | --- | --- | --- |
| *TP53* R249S mutation | p53^ser246^/HBs+ mice treated with AFB1 | C57Bl/6J | 13 months | PMID: 9537435 |
| *Trp53*-deletion | G4 mTERC^-/-^ /Trp53^F2-F10/F2-F10^ HBs+ mice treated with Ad-Cre | C57Bl/6J | 12-15 months | PMID: 17433324 |
| *TP53* R337H mutation | p53^R334H^ mice treated with DEN | C57Bl/6J | 42 weeks | PMID: 30042151 |

**Supplementary Table S5. List of murine HCC models with engineered p53.**
