# Supplementary material for: Loss of TP53 cooperates with c-MET overexpression to drive hepatocarcinogenesis
Source: Cell Death Dis. 2023 Jul 27;14(7):476. doi: 10.1038/s41419-023-05958-y (PMC10374654; doi:10.1038/s41419-023-05958-y)
Supplement: Supplementary file 7 — Supplementary Table S6 [file 41419_2023_5958_MOESM7_ESM.docx]

**Supplementary Table S6. List of human HCC cell lines used in this study.**

| **Cell lines** | **c-MET activation** | **TP53 Alteration type** | **Description** |
| --- | --- | --- | --- |
| LM9 | Yes | Mutation | E51*, E12* |
| HLE | Yes | Mutation | R249S |
