# Supplementary material for: Loss of TP53 cooperates with c-MET overexpression to drive hepatocarcinogenesis
Source: Cell Death Dis. 2023 Jul 27;14(7):476. doi: 10.1038/s41419-023-05958-y (PMC10374654; doi:10.1038/s41419-023-05958-y)

**Figure S15** Original western blots for Figure 2C


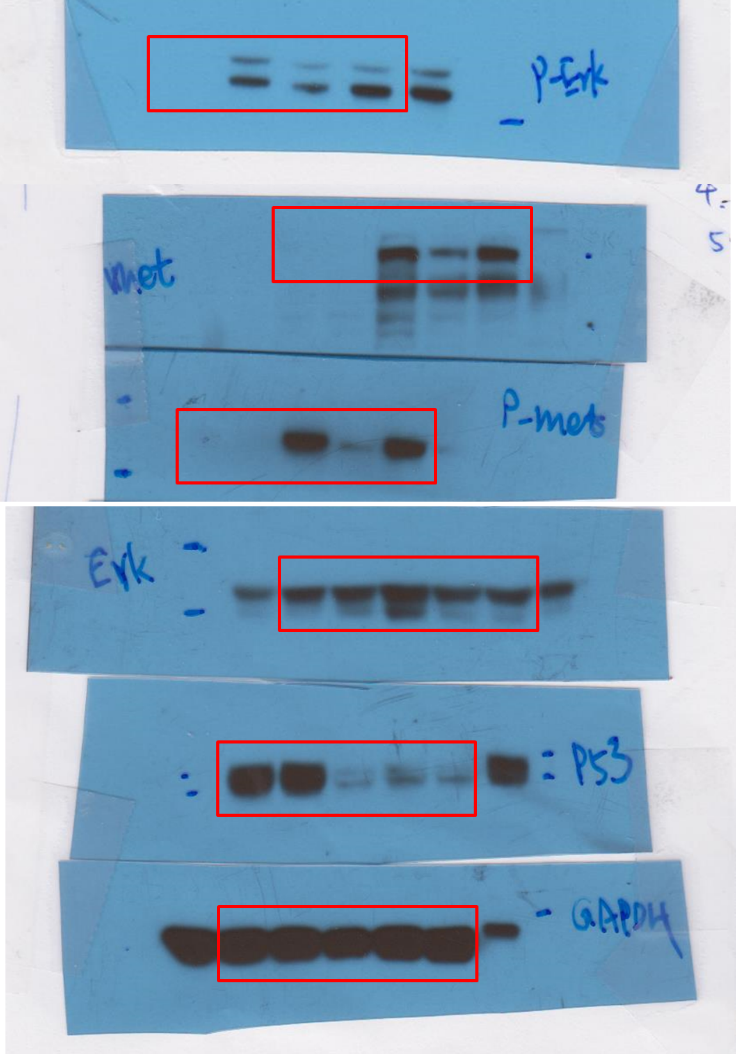


**Figure S16** Original western blots for Figure 4A


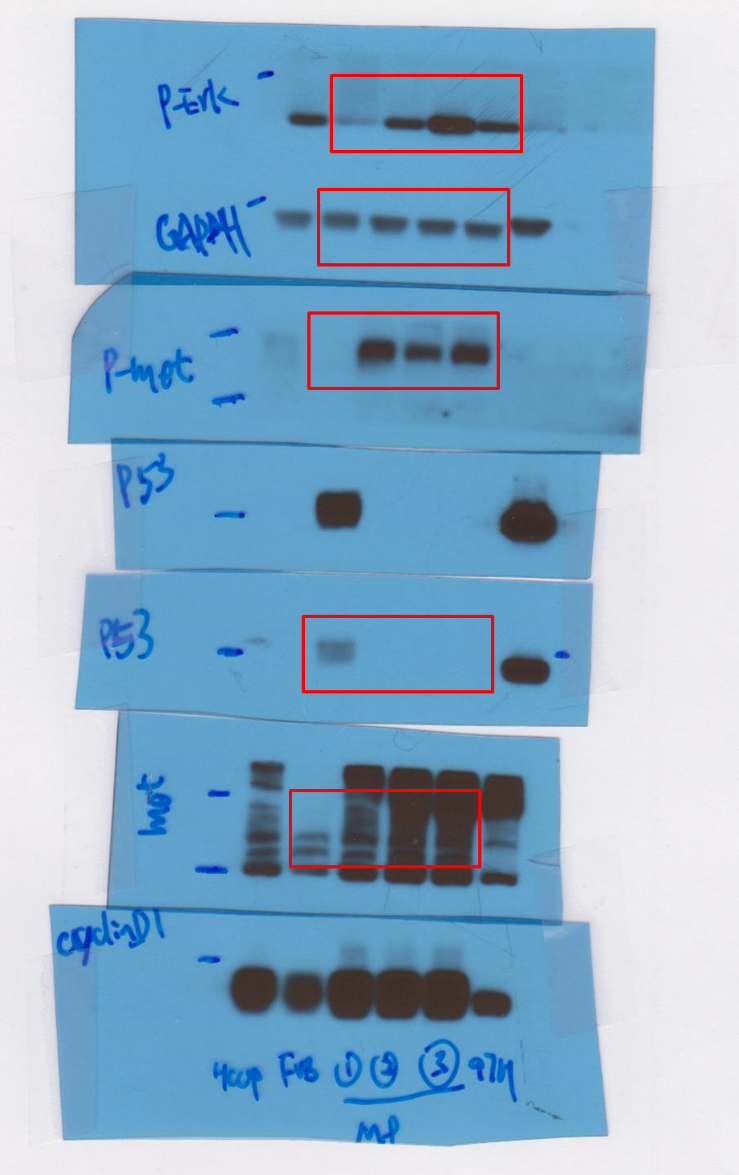


**Figure S17** Original western blots for Figure S14


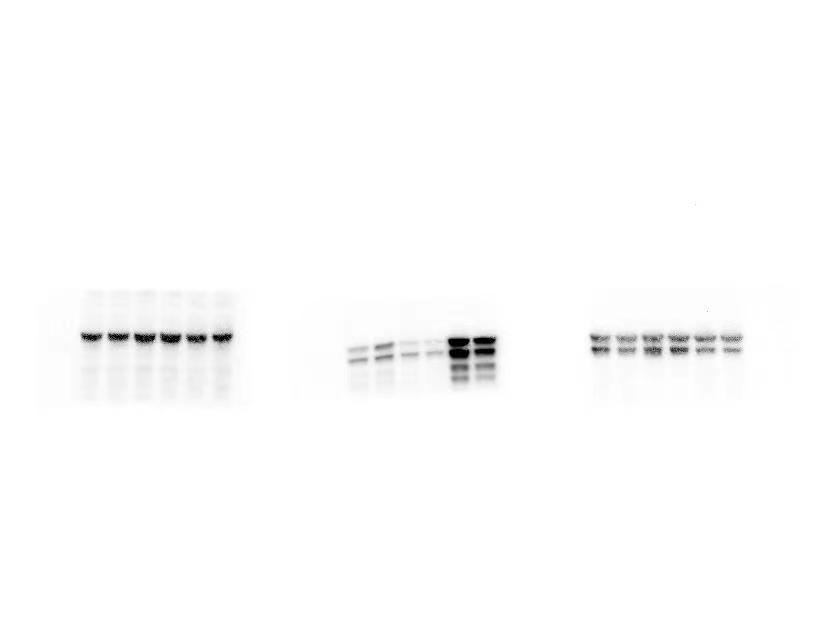

Supplement: Supplementary file 8 — Original western blots [file 41419_2023_5958_MOESM8_ESM.docx]
